# Effect of Specimen Dimensions and Strain Rate on the Longitudinal Compressive Strength of *Chimonobambusa utilis*

**DOI:** 10.3390/ma18174013

**Published:** 2025-08-27

**Authors:** Xudan Wang, Meng Zhang, Chunnan Liu, Bo Xu, Wei Li, Yonghong Deng, Yu Zhang, Chunlei Dong, Qingwen Zhang

**Affiliations:** 1 College of Civil Engineering, Southwest Forestry University, Kunming 650224, China; wxd9919@swfu.edu.cn (X.W.); zmeng@swfu.edu.cn (M.Z.); lcn9025@swfu.edu.cn (C.L.); 2 Resource Survey and Research Center, Yunnan Institute of Forest Inventory and Planning, Kunming 650051, China; 13759142496@163.com (B.X.); 18987350868@163.com (W.L.); dyh_55@163.com (Y.D.); zhangyu349@outlook.com (Y.Z.); 3 College of Materials and Chemical Engineering, Southwest Forestry University, Kunming 650224, China; dchunlei@swfu.edu.cn

**Keywords:** small-diameter bamboo, longitudinal compressive strength, Weibull model, strain rate sensitivity, coupled effect

## Abstract

The combined influence of specimen size and strain rate on the mechanical behaviour of small-diameter bamboo culms remains insufficiently characterised. This study investigates the longitudinal compressive strength of *Chimonobambusa utilis* through axial compression tests on specimens measuring 15 × 15 × 5 mm, 18 × 18 × 6 mm, and 21 × 21 × 7 mm under strain rates of 10^−4^, 10^−3^, and 10^−2^ s^−1^. Coupling experimental data with theoretical analysis, this study develops a size–strain rate interaction model to quantitatively assess the effects of specimen size and strain rate on the compressive strength of small-diameter bamboo. Increasing specimen size reduced strength and shifted failure modes from shear to buckling and splitting. At a strain rate of 10^−4^ s^−1^, strength decreased from 73.35 MPa for the 15 × 15 × 5 mm specimens to 62.84 MPa for the 21 × 21 × 7 mm specimens. Conversely, increasing the strain rate from 10^−4^ s^−1^ to 10^−2^ s^−1^ for the 15 × 15 × 5 mm specimens increased strength from 73.35 MPa to 80.27 MPa, indicating suppressed crack propagation. The Type II Weibull model exhibited higher predictive accuracy and parameter stability than the Type I variant. Coupling the Type II Weibull function with a power-law strain rate term and an interaction exponent developed a predictive equation, achieving relative errors below 5%. The findings demonstrate that specimen size predominantly governs strength, whereas strain rate exerts a secondary but enhancing influence. The proposed coupling model enables reliable axial load prediction for small-diameter bamboo culms, supporting material selection and dimensional optimisation in structural applications.

## 1. Introduction

Bamboo is widely recognised for its combination of high specific strength, renewability, and environmental compatibility, making it a material of considerable potential in lightweight structural systems [1,2], temporary building applications [3], and earthquake-resistant constructions [4]. Nevertheless, the natural variability and pronounced anisotropy inherent to bamboo result in mechanical behaviour that is significantly affected by parameters such as specimen size, loading orientation, and strain rate, with the size and strain rate effects playing a particularly critical role in structural design and reliability assessment.

In terms of the size effect of specimens, the longitudinal compressive strength decreases with increasing cross-sectional dimension: doubling the section size results in an approximate 2–5% reduction in strength, accompanied by fibre buckling and localised crushing [5,6]; similarly, when specimen length increases from 0.87 m to 1.17 m, the ultimate bending strength declines by approximately 11.38% [7]. This behaviour can be interpreted within the framework of the weakest-link concept, in which the Weibull theory, grounded in extreme value statistics, models a material as an assembly of multiple segments or units whose overall strength is governed by the weakest element [8]. Building upon this framework, size effect models incorporating the spatial distribution of defects have been developed and are now widely applied in material strength evaluation and structural reliability analysis [9,10].

Regarding the strain rate effect, the mechanical properties of materials vary systematically with changes in the applied strain rate [11,12,13,14]. Experimental studies have shown that, in both longitudinal and transverse directions, the strength and ultimate strain of bamboo increase with rising strain rate; when the strain rate is expressed on a logarithmic scale, the dynamic increase factor (DIF) exhibits a bilinear relationship, with inflection points at approximately 689 s^−1^ (longitudinal) and 525 s^−1^ (transverse). Below these points, the rate of increase is moderate, whereas above them, the enhancement becomes pronounced, with the transverse direction showing greater sensitivity [14]. Split Hopkinson pressure bar (SHPB) compression tests have further revealed that the yield stress parallel to the fibres increases from 38.59 MPa to 94.25 MPa. In comparison, that perpendicular to the fibres rises from 7.18 MPa to 42.04 MPa, accompanied by a transition in failure mode from vascular bundle buckling and parenchyma collapse to vascular bundle fracture and the formation of multiple shear bands, together with pronounced interfacial debonding at the microscale [15].

In modelling the strain rate effect, notable differences exist among available formulations. The Johnson–Cook model quantifies a material’s sensitivity to strain rate by introducing the strain rate sensitivity coefficient, *C* [16]. Previous studies [15,17] have indicated that although the degree of strengthening in bamboo is limited within the low-to-moderate strain rate range, retaining the strain rate term in the model is beneficial for capturing the trend of strength variation with loading rate. Fitting analyses conducted for natural fibre composites further demonstrated that a power-law formulation is superior in describing the strength–strain rate relationship [18]. Accordingly, the present study adopts a power-law model to characterise the strain rate effect of small-diameter bamboo and couples it, within a unified linearised framework, with the size effect of specimen model through regression analysis.

Among various small-diameter bamboo species, *Chimonobambusa utilis* is a medium-diameter sympodial bamboo with a well-developed multi-culm rhizome system, deriving its common name from its characteristic quadrangular culm cross-section [19]. In mature culms aged 3–5 years, the basal wall thickness ranges from 4 to 9 mm, each internode measures approximately 20–30 cm in length, and setae are present along the node regions. In Southwest China, the natural distribution range, ecological dominance, and resource abundance of *Chimonobambusa utilis* rank second only to *Chimonobambusa tumidissinoda* [20]. Due to its significance as a natural resource and its representativeness among small-diameter bamboo species, *Chimonobambusa utilis* was selected as the subject of this study.

Although previous studies have examined the size effect of specimens and the strain rate effect of bamboo, systematic research on their coupled interaction and corresponding mechanical modelling remains limited. The existing literature has predominantly focused on large-diameter bamboo, addressing the influence of individual factors on strength, with relatively few analyses dedicated to small-diameter species. To address this gap, the present study investigates *Chimonobambusa utilis* through longitudinal compression testing, develops a coupled size–strain rate model, and elucidates the combined influence of these parameters on longitudinal compressive strength. The objective is to improve the predictive accuracy of mechanical performance and provide a theoretical basis for structural design and reliability assessment of small-diameter bamboo in green building applications.

## 2. Materials and Methods

### 2.1. Materials

Specimens were prepared from 3–5-year-old *Chimonobambusa utilis* harvested from the same batch in Yanjin County, Zhaotong, Yunnan Province (104.17° E, 28.39° N). Sourcing all specimens from a single population minimized material variability and ensured the reliability of the results.

**Figure 1:**
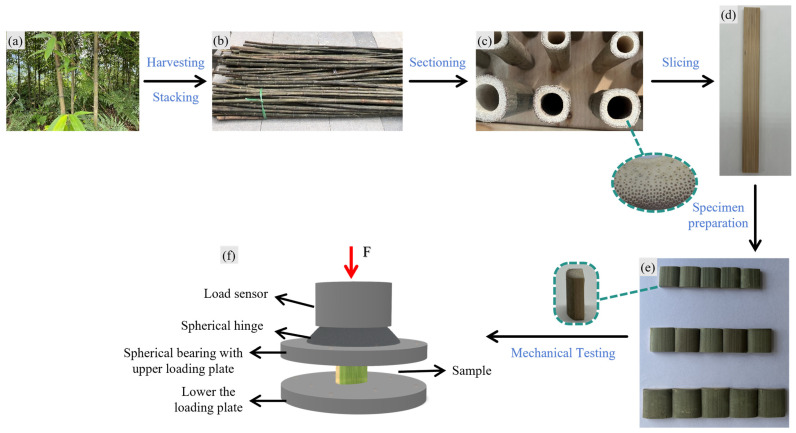
Preparation procedure of bamboo specimens with different dimensions: (**a**) Harvesting of *Chimonobambusa utilis*; (**b**) stacking; (**c**) cutting into sections of the required length, with the dashed circle on the right highlighting an enlarged view of a single thin flake specimen after slicing; (**d**) longitudinal bamboo flake; (**e**) three prepared flaky specimens (15 × 15 × 5 mm, 18 × 18 × 6 mm, and 21 × 21 × 7 mm, with the dashed outline on the left indicating the specimen’s elevation view); (**f**) schematic diagram of longitudinal compressive loading test.

Specimens were processed in accordance with the Test Methods for the Physical and Mechanical Properties of Bamboo Materials for Construction (JG/T 199−2007) [21], using a GTS10J table saw (Robert Bosch GmbH, Zhejiang, China) and an FM15901 planing machine (Fengmo Tools, Beijing, China), followed by trimming with a bamboo knife. Final dimensions were verified using a digital calliper (Mitutoyo Corporation, Guangdong, China, accuracy ± 0.01 mm). Three groups of *Chimonobambusa utilis* specimens with varying dimensions (width × height × thickness: 15 mm × 15 mm × 5 mm, 18 mm × 18 mm × 6 mm, and 21 mm × 21 mm × 7 mm) were prepared for longitudinal compression testing, as illustrated in Figure 1. The specimens were first air-dried for seven days in a ventilated environment and then oven-dried (using an oven from Shanghai Yuejin Medical Equipment Co., Ltd., Shanghai, China) to a moisture content of ≤12% to ensure consistency during testing.

Moisture content (*MC*) was determined using the oven-drying method, as specified in JG/T 199−2007 [21]. For each group, three specimens were randomly selected and weighed using a precision electronic balance (model: JA3002G, Shanghai Xiniu Technology Co., Ltd., Shanghai, China) with an accuracy of ±0.01 g. Samples were dried at (103 ± 2) °C and reweighed every 24 h until the mass change was within 0.1%, at which point the oven-dry mass was recorded. The moisture content was calculated using Equation (1):

(1) $$MC=\frac{m_{1}-m_{2}}{m_{2}}\times 100\%$$

where *m*_1_ and *m*_2_ represent the initial mass and the oven-dried mass, respectively.

### 2.2. Test Method

#### 2.2.1. Longitudinal Compression Test

The longitudinal compression test was conducted in accordance with the Test Methods for the Physical and Mechanical Properties of Bamboo Materials for Construction (JG/T 199−2007) [21], using a Shimadzu universal testing machine (Model: UTM5105) manufactured by Shenzhen Sansizongheng Technology Co., Ltd. (Shenzhen, China) and equipped with a 10 kN load cell (Model: 10kNSX) to measure the applied force.

Three strain rate levels were applied: 10^−4^ s^−1^, 10^−3^ s^−1^, and 10^−2^ s^−1^. The target strain rate ($\dot{\varepsilon }$) was calculated using Equation (2):

(2) $$\varepsilon =\frac{V}{L_{0}}$$

where *V* is the loading speed (mm/min), and *L*_0_ is the specimen height.

Table 1 lists the loading speeds required to achieve each target strain rate for the three specimen heights.

Each group consisted of nine specimens, totalling 81 specimens across all test conditions. To minimize friction, a thin layer of Vaseline was evenly applied to both ends of the specimens before loading. Based on the mechanical test data obtained using the universal testing machine, the longitudinal compressive strength and elastic modulus were calculated according to Equations (3) and (4), respectively.

(3) $$f=\frac{P_{max}}{bt}$$

(4) $$E=\frac{20\Delta P}{bt\Delta l}$$

In the equations, *f* represents the longitudinal compressive strength; *P*_max_ is the failure load; *b* is the specimen width; *t* is the specimen thickness; *E* is the longitudinal compressive elastic modulus; ΔP is the difference between the upper and lower limit loads; and Δl is the difference in specimen deformation under the upper and lower limit loads.

Surface strain evolution was recorded using digital image correlation (DIC) with the LINCONST 5M/5MPRO system, manufactured by Shenzhen SansiZongheng Technology Co., Ltd., in Shenzhen, China. A speckle pattern was applied to the specimen surface, and images were captured at a rate of three frames per second. The test was terminated when visible cracks appeared or the load dropped to 70% of the peak value.

#### 2.2.2. Microstructural Observation

To examine the microstructural characteristics of *Chimonobambusa utilis* under different failure modes, the morphology of vascular bundles was observed using a Zeiss GeminiSEM 360 field-emission scanning electron microscope (Zeiss, Oberkochen, Germany). Specimens were sampled from uncompressed bamboo and typical failure zones following longitudinal compression. After sampling, the specimens were oven-dried to a moisture content of ≤12% and then subjected to gold sputter coating for approximately 60 s to enhance electrical conductivity.

The scanning electron microscopy (SEM) observations were conducted at an accelerating voltage of 3–5 kV, under a vacuum level of 2 × 10^−3^ Pa in high-vacuum mode. Magnifications of 300× and 1000× were used, corresponding to scale bars of 30 μm and 10 μm, respectively. Images were captured in secondary electron (SE) mode to characterize the vascular bundle structures associated with different failure modes.

### 2.3. Theoretical Analysis of Size and Strain Rate Effects

#### 2.3.1. Weibull Weakest-Link Theory

Based on the Weibull weakest-link theory, the relationship between material strength and specimen size was established, as expressed in Equation (5):

(5) $$\frac{f_{0}}{f}={\left(\frac{V}{V_{0}}\right)}^{S}$$

where *V* and *V*_0_ are the volumes of the test and reference specimens, *f* and *f*_0_ are the corresponding strengths, and *S* is the size effect of specimen coefficient.

To account for the combined influence of width, height, and thickness on bamboo, a size effect coefficient was introduced, enabling Equation (5) to be transformed into Equation (6):

(6) $$\frac{f_{0}}{f}={\left(\frac{V}{V_{0}}\right)}^{S}={\left(\frac{b}{b_{0}}\times \frac{l}{l_{0}}\times \frac{t}{t_{0}}\right)}^{s}$$

When the height-to-thickness ratio is constant, Equation (6) can be converted into Equation (7):

(7) $$\frac{f_{0}}{f}={\left(\frac{b}{b_{0}}\times \frac{l}{l_{0}}\times \frac{t}{t_{0}}\right)}^{s}={\left(\frac{b}{b_{0}}\times \frac{l}{l_{0}}\right)}^{s}={\left(\frac{b}{b_{0}}\times \frac{\tau b}{{\tau b}_{0}}\right)}^{s}={\left(\frac{b^{2}}{b_{0}^{2}}\right)}^{S_{\tau }}={\left(\frac{b_{0}^{2}}{b^{2}}\right)}^{-S_{\tau }}$$

where *V* and *V*_0_ denote the volumes of the current and reference specimens, *f* and *f*_0_ are the corresponding strengths, *b* and *b*_0_ are the thicknesses, *l* and *l*_0_ are the heights, *t* and *t_0_* are the widths, and *S_τ_* is the size effect of the specimen exponent associated with the height-to-thickness ratio, derived based on the Weibull weakest-link theory.

Using linear regression analysis, the size effect of the specimen parameter is determined, and Equation (7) can be further simplified into the linear regression form shown in Equation (8):

(8) $$\log\left(\frac{f_{0}}{f}\right)=-S_{\tau }\log\left(\frac{b_{0}^{2}}{b^{2}}\right)=S_{\tau }\log\left(\frac{b^{2}}{b_{0}^{2}}\right)$$

Equation (8) is designated as the Type I Weibull size effect model and is employed for the subsequent calculation of size effect parameters. The size effect of the specimen exponent is determined through linear regression analysis of the measured longitudinal compressive strength and the cross-sectional area ratio. The fitted linear regression equation is presented as Equation (9):

(9) y=mx+c

where $m=S_{\tau }$; $y=\log\left(\frac{f_{0}}{f}\right)$; $x=\log\left(\frac{b^{2}}{b_{0}^{2}}\right)$.

Assuming that the material strength follows a three-parameter Weibull distribution, the relationship between longitudinal compressive strength and specimen volume *V* can be obtained and is defined as the Type II model, with the corresponding expression given in Equation (10).

(10) $$\sigma_{N}=\sigma_{0}{\left(\frac{D}{D_{0}}\right)}^{-m}$$

where *σ_N_* is the nominal longitudinal compressive strength at the specimen’s characteristic dimension *D*, *σ*_0_ is the reference strength at size *D*_0_, and *m* is the size effect of the specimen exponent, which characterizes the scale-dependent reduction in strength due to flaw accumulation.

The parameter *m* reflects the influence of flaw distribution as interpreted through the weakest-link framework and is theoretically analogous to the scale parameter in a Weibull distribution.

#### 2.3.2. Strain Rate Effect

The standard form of the Johnson–Cook model comprises a strain hardening term, a strain rate effect term, and a thermal softening term, as expressed in Equation (11).

(11) $$\sigma =\left(A+B\varepsilon^{n}\right)\left[1+C\ln\frac{\dot{\varepsilon }}{\dot{\varepsilon_{0}}}\right]\left[1-{\left(T^{*}\right)}^{m}\right]$$

where *σ* is the longitudinal compressive strength (MPa), *ε* denotes the equivalent plastic strain, $\dot{\varepsilon }$ is the applied strain rate, $\dot{\varepsilon_{0}}$ is the reference strain rate, *T* represents the homologous temperature, and *A*, *B*, *C*, *n*, and *m* are material constants.

For longitudinal bamboo compression, the failure mode is predominantly brittle fracture, with no distinct stage of plastic deformation and no significant strain hardening or thermal softening observed within the experimental range; therefore, the model can be simplified and expressed as Equation (12).

(12) $$\sigma =\sigma_{0}\left[1+C\ln\frac{\dot{\varepsilon }}{\dot{\varepsilon_{0}}}\right]$$

where *σ* is the longitudinal compressive strength at the applied strain rate $\dot{\varepsilon }$, *σ*_0_ is the reference strength at strain rate $\dot{\varepsilon_{0}}$, and *C* is the strain rate sensitivity exponent.

After taking the logarithm, the equation can be transformed into a power-law form, as shown in Equation (13):

(13) $$\sigma =\sigma_{0}{\left(\frac{\dot{\varepsilon }}{\dot{\varepsilon_{0}}}\right)}^{n}$$

where *σ* is the longitudinal compressive strength at strain rate $\dot{\varepsilon }$, *σ*_0_ is the reference strength at strain rate $\dot{\varepsilon_{0}}$, and *n* denotes the strain rate sensitivity index, which quantifies the strengthening effect induced by strain rate-dependent microscopic failure mechanisms.

#### 2.3.3. Establishment of the Coupled Model

To simultaneously account for the effects of size and strain rate on the longitudinal compressive strength of *Chimonobambusa utilis*, a coupled model is established by introducing the characteristic size *D*_0_ and the reference strain rate $\dot{\varepsilon_{0}}$. Equation (14) represents the coupling of the Type I Weibull size effect model with the strain rate model, whereas Equation (15) represents the coupling based on the Type II model:

(14) $$\sigma =\sigma_{0}{\left(\frac{D}{D_{0}}\right)}^{-S_{\tau }}{\left(\frac{\dot{\varepsilon }}{\dot{\varepsilon_{0}}}\right)}^{n}$$

(15) $$\sigma =\sigma_{0}{\left(\frac{D}{D_{0}}\right)}^{-m}{\left(\frac{\dot{\varepsilon }}{\dot{\varepsilon_{0}}}\right)}^{n}$$

where *σ* denotes the longitudinal compressive strength under specimen size *D* and strain rate $\dot{\varepsilon }$, while *σ*_0_ represents the reference strength at size *D*_0_ and strain rate $\dot{\varepsilon_{0}}$. The parameter *S_τ_* is the size effect of the specimen exponent in the Type I model. The exponent *m* corresponds to the size effect of the specimen in the Type II model. The parameter *n* is the strain rate sensitivity exponent.

Under the range of testing conditions, these coupled models were employed to fit longitudinal compressive strength values across different combinations of specimen sizes and strain rates. This fitting process enabled the characterization of underlying trends and facilitated cross-scale prediction of longitudinal compressive performance in small-diameter bamboo.

## 3. Experimental Results

### 3.1. Failure Characteristics

#### 3.1.1. Macroscopic Structure

**Figure 2:**
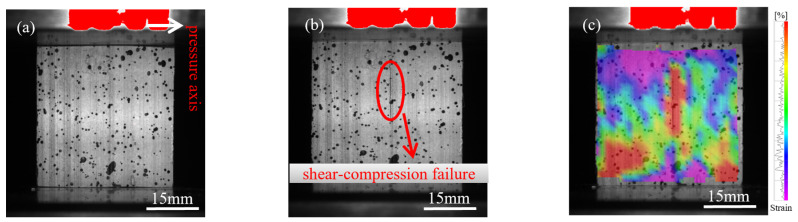
Failure progression of the 15 mm specimen under dynamic compression: (**a**) initial compression stage; (**b**) shear-compression failure; (**c**) strain field illustrating shear failure (DIC).

**Figure 3:**
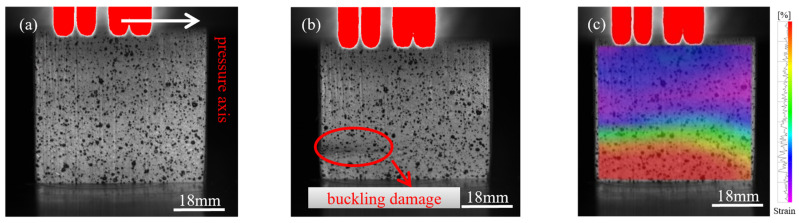
Failure progression of the 18 mm specimen under dynamic compression: (**a**) initial compression stage; (**b**) buckling failure; (**c**) strain field illustrating buckling failure (DIC).

**Figure 4:**
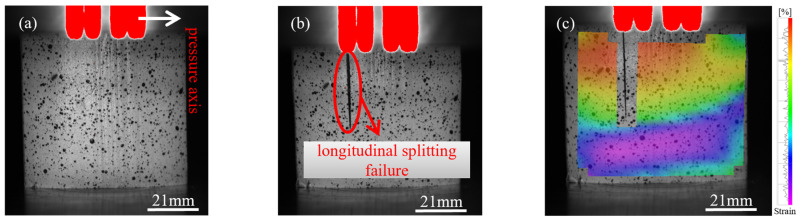
Failure progression of the 21 mm specimen under dynamic compression: (**a**) initial compression stage; (**b**) splitting failure; (**c**) strain field illustrating splitting failure (DIC).

During longitudinal compression testing of bamboo specimens, progressive changes in failure modes were observed with increasing specimen size. These transitions ranged from shear-compression failure to buckling and ultimately splitting failure (Figure 2, Figure 3 and Figure 4). The size effect of the specimen results in a non-uniform stress distribution, whereby larger specimens are more prone to local buckling or crack propagation during loading, further intensifying the failure mode’s complexity.

Figure 2a shows the initial loading state of the 15 mm specimen, followed by shear-compression failure in Figure 2b. This failure mode is attributed to localized regions experiencing simultaneous compression and shear, caused by non-uniform internal stress distribution. Buckling failure occurred in the 18 mm specimen, as shown in Figure 3a,b, due to structural instability when the axial load exceeded the critical buckling threshold. Figure 4a illustrates the initial loading phase of the 21 mm specimen, and Figure 4b shows the splitting failure caused by uneven compressive stress distribution across the cross-section.

Figure 2c, Figure 3c, and Figure 4c present the failure stress contour maps for the 15 mm, 18 mm, and 21 mm specimens, respectively. The colour bar on the right indicates the correspondence between colour and stress magnitude: purple-blue regions represent stress levels below 20 MPa, while red regions denote areas where stress exceeds 60 MPa. The contour plots clearly show how stress distribution varies under different failure modes. Specifically, shear-compression failure is characterized by diagonally oriented stress bands; buckling failure exhibits localized zones of axial instability; and splitting failure is marked by radially propagating cracks. The alignment between the stress contours and the observed macroscopic failure modes further elucidates the evolution of failure mechanisms as influenced by specimen size.

#### 3.1.2. Microstructural Evolution Under Different Failure Modes

**Figure 5:**
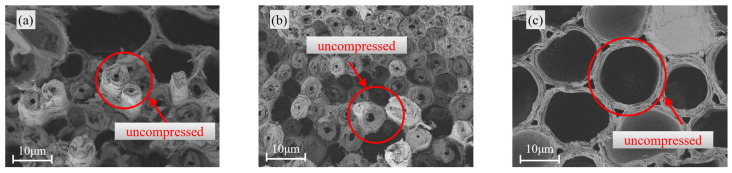
Original vascular bundle morphology of *Chimonobambusa utilis*: (**a**) 15 mm; (**b**) 18 mm; (**c**) 21 mm.

**Figure 6:**
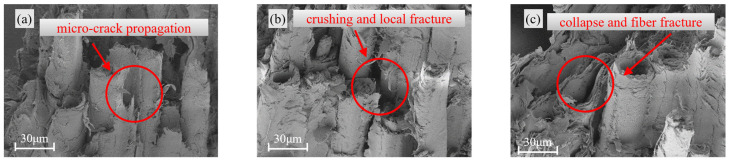
Vascular bundle morphology under buckling failure: (**a**) 15 mm; (**b**) 18 mm; (**c**) 21 mm.

**Figure 7:**
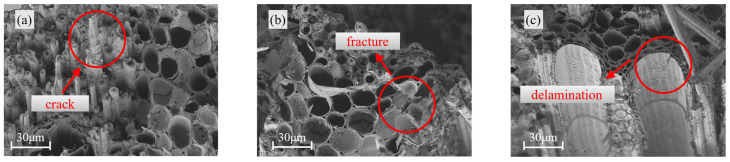
Vascular bundle morphology under splitting failure: (**a**) 15 mm; (**b**) 18 mm; (**c**) 21 mm.

Figure 5, Figure 6 and Figure 7 present the morphological characteristics of vascular bundles in bamboo under three conditions: the uncompressed state, regions exhibiting buckling failure after longitudinal compression, and regions affected by splitting failure.

The microstructure of bamboo exhibited notable variability with increasing specimen size (Figure 5, Figure 6 and Figure 7). In the uncompressed state (Figure 5), the vascular bundles of the 15 mm specimen maintained an intact morphology, with smooth and continuous inner walls and no visible cracks or structural defects. However, in the 21 mm specimen, microcracks began to form within the vessel walls. Localized voids and microcracks were particularly evident along the vessel interfaces, indicating the onset of internal damage as specimen size increased.

Under buckling failure conditions (Figure 6), distinct deformation features were observed across specimens of different sizes. The 21 mm specimen displayed pronounced collapse of vessel walls and disordered alignment of vascular bundles, whereas the 15 mm specimen largely retained a coherent structural configuration. These morphological differences suggest that larger specimens are more prone to local instability or collapse induced by stress concentration during compressive loading.

Microstructural observations in the splitting failure region (Figure 7) further confirmed the influence of specimen size on damage evolution. In the 21 mm specimen, extensive fracture and delamination of vascular bundles were observed. In contrast, the 18 mm and 15 mm specimens exhibited more localized damage, with only partial fibre breakage present. This progressive damage pattern reinforces the conclusion that increasing specimen size intensifies the severity and extent of structural failure in bamboo.

These observations are consistent with previous studies on structural and reconstituted bamboo [5,6], indicating that with increasing specimen size, damage phenomena such as fibre buckling, localised crushing, and crack propagation become more pronounced. Moreover, the probability of exposing internal defects increases significantly with size, leading to a transition in failure mode from a single shear-dominated pattern to more complex multi-crack propagation and splitting patterns. The specimen size effect revealed through the microstructural observations in this study further confirms the generality of the influence of increasing dimensions on the failure modes of bamboo.

### 3.2. Load–Displacement Behaviour

**Figure 8:**
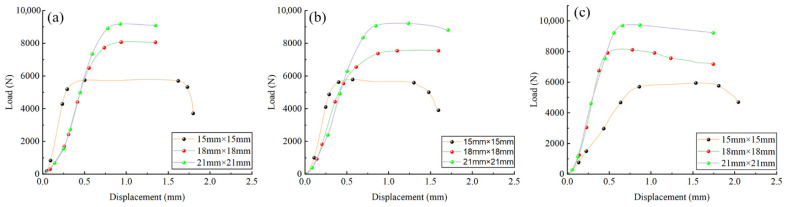
Displacement–load curves of *Chimonobambusa utilis* specimens with varying sizes under different strain rates: (**a**) strain rate of 10^−4^ s^−1^; (**b**) strain rate of 10^−3^ s^−1^; (**c**) strain rate of 10^−2^ s^−1^.

**Figure 9:**
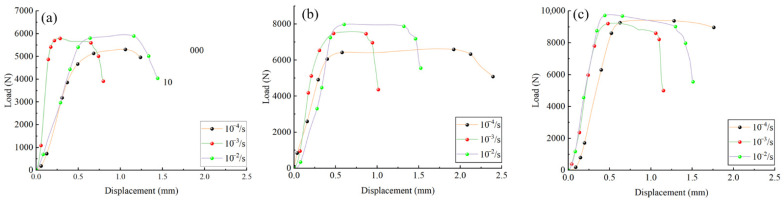
Displacement–load curves of *Chimonobambusa utilis* specimens with different strain rates at fixed sizes: (**a**) 15 mm × 15 mm; (**b**) 18 mm × 18 mm; (**c**) 21 mm × 21 mm.

The mean values of each group of specimens were used to plot the load–displacement curves under different strain rates and specimen sizes, as shown in Figure 8 and Figure 9. Coloured dots in the figures mark key points selected based on distinct changes in load and displacement, illustrating the differences exhibited by bamboo specimens of various sizes and strain rates during loading.

Figure 8 and Figure 9 show that the load–displacement relationships of the specimens vary markedly with specimen size and strain rate. For specimens measuring 15 × 15 mm, the slope of the curve is higher, indicating greater stiffness, under low strain rates of 10^−4^ s^−1^ and 10^−3^ s^−1^. When the strain rate increases to 10^−2^ s^−1^, the slope decreases substantially, reflecting a reduction in stiffness. This reduction is associated with the rapid initiation and propagation of microcracks within the bamboo at high strain rates, which weakens the stress transfer between fibres and cell walls, shortens the elastic deformation stage, and leads to the earlier onset of inelastic deformation. The influence of microcracks on the overall mechanical performance is more pronounced in smaller specimens, resulting in a more significant stiffness loss. Overall, the load exhibits a nonlinear increase with displacement; at the same strain rate, the maximum load increases with specimen size, whereas at the same size, a higher strain rate produces a greater increase in load.

Table 2 presents the test data results. Within the strain rate range of 10^−4^ s^−1^ to 10^−2^ s^−1^, the longitudinal compressive strength parallel to the grain decreases with increasing specimen size; at the same size, longitudinal compressive strength increases with increasing strain rate.

The values in the table represent the mean results for each specimen group and are used for descriptive statistics. A two-way analysis of variance (ANOVA) was performed using the original measured values of each specimen, and the results indicated that the effect of size on longitudinal compressive strength was significant (*p* < 0.01). In contrast, the influence of strain rate within the experimental range did not reach a statistically significant level (*p* > 0.05). Analysis of mean trends revealed that, under different size conditions, the strength of specimens increased to varying degrees with higher loading rates, consistent with theoretical predictions. These findings indicate that the specimen size effect is the dominant factor. At the same time, the strain rate exerts a moderating and enhancing influence on strength variation within a specific range, with a trend of coupling between the two factors.

Simultaneously, data from the table indicate that specimens measuring 15 × 15 × 5 mm tested under a strain rate of 10^−3^ s^−1^ exhibited the highest elastic modulus along with relatively high longitudinal compressive strength; however, the coefficient of variation was significant, implying that higher stiffness is accompanied by greater performance variability. Under the 10^−2^ s^−1^ strain rate, specimens showed a comparatively lower elastic modulus but a smaller standard deviation and coefficient of variation, suggesting that material performance is more stable at higher strain rates, albeit with a reduction in strength.

**Figure 10:**
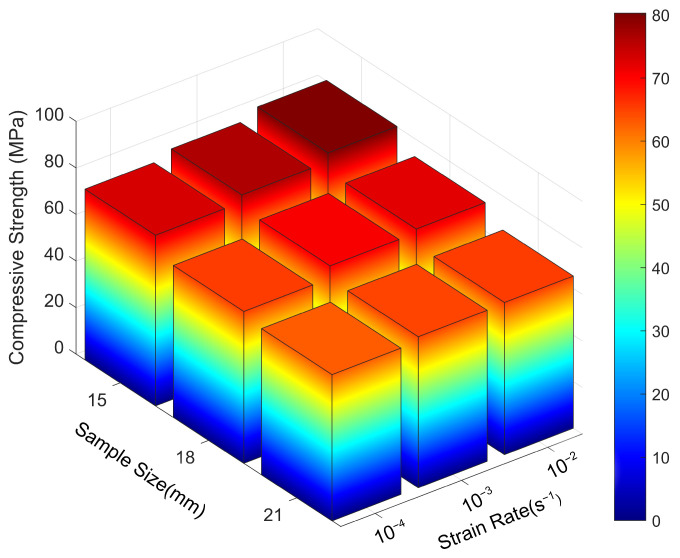
Three-dimensional distribution of longitudinal compressive strength under the combined effects of specimen size and strain rate.

Figure 10 illustrates the three-dimensional distribution of longitudinal compressive strength as influenced by the combined effects of specimen size and strain rate. The colour bar on the right represents the mapping between longitudinal compressive strength values and corresponding colours, facilitating direct visualisation of strength variation trends.

With increasing strain rate, the longitudinal compressive strength of specimens across all sizes increased progressively. This trend is visually represented by a colour shift from blue to red along the vertical axis, indicating enhanced compressive capacity at higher strain rates. At a constant strain rate, a negative correlation was observed between specimen size and longitudinal compressive strength: larger specimens exhibited lower strength values and correspondingly lighter colour intensity. These results underscore the pronounced influence of the specimen size effect on compressive performance along the fibre direction.

### 3.3. Theoretical Model and Fitting Results

#### 3.3.1. Weibull Weakest-Link Theory—Type I

**Figure 11:**
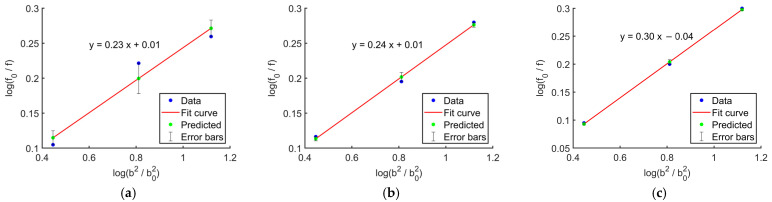
Fitting curves of size effect parameters at different strain rates: (**a**) strain rate 10^−4^ s^−1^; (**b**) strain rate 10^−3^ s^−1^; (**c**) strain rate 10^−2^ s^−1^.

Figure 11 presents the linear regression results for the relationship between longitudinal compressive strength and specimen size of *Chimonobambusa utilis* under three strain rates (10^−4^ s^−1^, 10^−3^ s^−1^, and 10^−2^ s^−1^). For a relatively small range of specimen sizes, the relationship between strength and size can be approximated as linear through logarithmic transformation, by the Weibull weakest-link theory. The three data points in the figure represent mean values, adopted to reduce the scatter caused by natural variability; since the within-group variation was minimal, a three-point fitting approach was employed in this study.

Figure 11 shows that the fitting curves for all three strain rates exhibit strong linearity, validating the applicability of the Weibull weakest-link theory Type I in describing the specimen size effect in bamboo. Based on the specimen size effect parameter *S_τ_* obtained from the Type I Weibull weakest-link theory, the predicted longitudinal compressive strengths for the three specimen sizes are presented in Table 3.

As shown in Table 3, within the strain rate range of 10^−4^ s^−1^ to 10^−2^ s^−1^, the prediction errors of the Weibull weakest-link theory Type I for specimens of different sizes remained within 5%. This confirms the model’s applicability and high fitting accuracy under low to intermediate strain rate conditions.

Further analysis revealed that the size effect parameter *S_τ_* increased from 0.23 to 0.30 with rising strain rate. This trend indicates an amplification of the influence of local defects on the material at higher strain rates. During rapid loading, microcracks or weakest links within the material cannot fully release stress, making larger specimens more susceptible to failure due to the accumulation of internal defects. The R^2^ values listed in the table are all close to 1, demonstrating that the weakest-link model can well-describe the longitudinal compressive strength failure mechanism. This consistency across varying specimen sizes and strain rates reflects the dominant role of microstructural defects in governing the macroscopic mechanical behaviour of the material.

#### 3.3.2. Weibull Weakest-Link Theory—Type II

Through comparative analysis, it was determined that selecting the intermediate size of 18 mm as the reference dimension (*D*_0_) provided more stable and representative fitting results. Therefore, this value was consistently adopted throughout the study.

**Figure 12:**
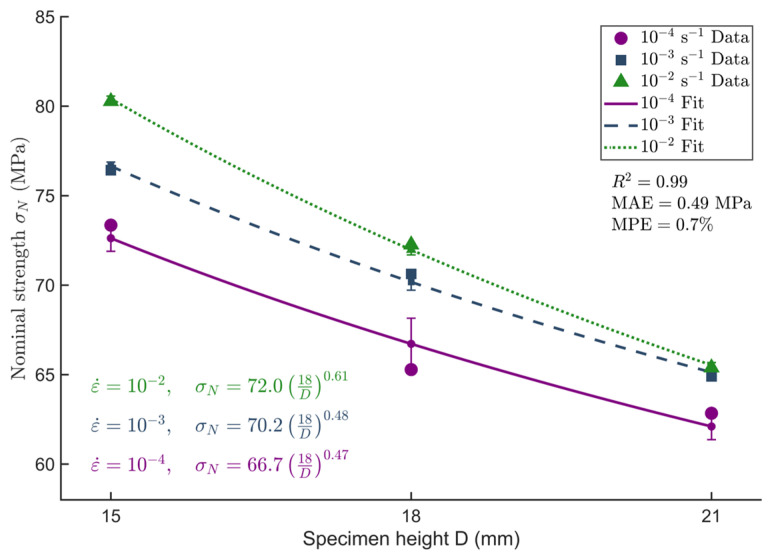
Fitting curves based on the Weibull weakest-link theory Type II.

The fitted values of *D*_0_, *m*, and *σ*_0_ were obtained via linear regression and are presented in Table 4. The corresponding fitting curves are shown in Figure 12.

The value of *m* rises from 0.47 to 0.61, suggesting that the strength distribution within the material becomes increasingly focused as strain rate increases. This implies that the effect of internal defects on strength is confined to a narrower range, concentrating failure on a limited number of weak spots, which reduces overall variability in strength. Nevertheless, the model’s predictive accuracy declines under extreme loading conditions, likely due to nonlinear deformation phenomena or other micro-mechanisms during high-rate loading that are not fully accounted for in the model.

To further evaluate the predictive capability of the Weibull weakest-link theory Type II for the longitudinal compressive strength of *Chimonobambusa utilis* under varying strain rates and specimen sizes, model fitting was conducted for each set of experimental data. The predicted values were then compared with the corresponding measured values, and both absolute and relative errors were calculated. The results are summarized in Table 5.

Table 5 demonstrates that the Weibull weakest-link theory Type II exhibits robust fitting performance across various strain rates and specimen sizes, with relative errors consistently maintained below 5%. Notably, the medium-sized specimens (18 × 18 × 6 mm) yielded the lowest fitting error, indicating superior adaptability to this size. In particular, at a strain rate of 10^−3^ s^−1^, the model achieved its highest accuracy, with a relative error as low as 0.10%. However, the fitting error increases with rising strain rate, indicating that the material’s mechanical behaviour becomes more complex at higher strain rates. Defect distribution remains relatively stable at lower strain rates, allowing the model to achieve more accurate predictions.

#### 3.3.3. Strain Rate Model

**Figure 13:**
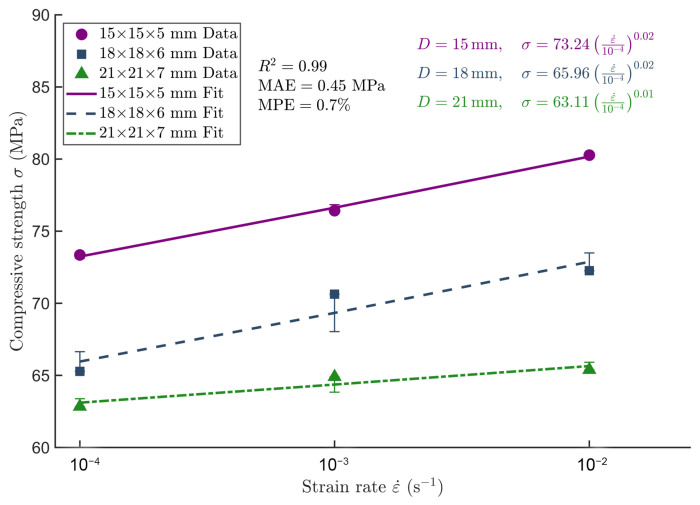
Fitting curves of strain rate sensitivity for *Chimonobambusa utilis* specimens of different sizes.

After evaluating the influence of different reference strain rates on model performance, the intermediate value was selected as the reference strain rate ($\dot{\varepsilon_{0}}$) to simplify the model formulation and enhance fitting stability. Accordingly, a reference strain rate of $\dot{\varepsilon_{0}}$ = 10^−3^ s^−1^ was adopted in this study. The fitted strain rate sensitivity parameters for each specimen size are summarized in Table 6, and the corresponding fitting results are illustrated in Figure 13.

The strain rate sensitivity exponent *n* decreases as specimen size increases, with smaller specimens exhibiting higher *n* values, indicating that the mechanical response of smaller specimens is more sensitive to changes in strain rate.

Figure 13 illustrates that the fitted curves for each specimen size agree well with the experimental data, confirming the applicability and accuracy of the proposed strain rate sensitivity model. A detailed comparison between the predicted and measured longitudinal compressive strength values based on this model is provided in Table 7.

According to Table 7, the prediction errors of the strain rate model for the longitudinal compressive strength of *Chimonobambusa utilis* were relatively low compared to the experimental data, with the maximum relative error not exceeding 1.76%. As the strain rate increased from 10^−4^ to 10^−2^ s^−1^, the prediction error did not show a significant increase, indicating that the model adequately captures the strain rate sensitivity and effectively reflects the material’s response variations under different loading rates.

## 4. Discussion

### 4.1. Mechanism of Size Effect on Mechanical Properties

At the macroscopic level, *Chimonobambusa utilis* exhibits a pronounced size effect under longitudinal compression, whereby longitudinal compressive strength decreases with increasing specimen size. The stress distribution and mechanical response vary across specimens of different dimensions, leading to distinct failure modes primarily governed by both size and strain rate. Buckling failure occurs when the applied axial load exceeds the critical threshold, while splitting failure is typically associated with non-uniform or locally concentrated stress conditions.

From a microscopic perspective, the likelihood of intrinsic defects—such as microcracks, voids, and interfibre inconsistencies—increases with specimen size. These imperfections reduce the effective load-bearing area, thereby diminishing the overall strength. SEM analysis revealed that larger specimens exhibited more severe deformation, delamination, and fracture of vascular bundles, indicative of intensified stress concentration regions during loading, which further amplify the specimen size effect.

### 4.2. Regulatory Effect of Strain Rate on Performance Response

Experimental findings indicate that increasing the strain rate enhances the longitudinal compressive strength of bamboo. At a strain rate of 10^−2^ s^−1^, the peak longitudinal compressive strength exhibited a marked increase across all specimen sizes. This improvement is primarily attributed to the fact that, under dynamic loading, the rate of stress propagation within the bamboo structure surpasses the rate of micro-damage development. This dynamic advantage promotes a more uniform internal stress distribution, thereby enhancing the material’s load-bearing capacity.

Furthermore, high strain rates inhibit the initiation and propagation of microcracks, allowing the material to sustain greater loads before failure. The combined influence of dynamic loading and stabilized internal stress fields significantly enhances the instantaneous load-bearing performance of *Chimonobambusa utilis*.

In Figure 8, the 18 mm and 21 mm specimens exhibited increasing stiffness with rising strain rate. In contrast, the 15 mm specimens demonstrated a gradual reduction in stiffness under higher strain rates, indicating that their stiffness decreases as the strain rate increases. This phenomenon may be associated with bamboo’s microstructural characteristics and viscoelastic behaviour. Microcrack propagation and local buckling in bamboo may become more pronounced at higher strain rates, resulting in stiffness degradation. Due to their more compact microstructure, smaller specimens are more susceptible to local instability. In contrast, larger specimens, with greater structural stability, can more effectively resist local deformation, thereby exhibiting enhanced stiffness. This phenomenon is consistent with the findings reported for balsa wood [22], in which lower stiffness was observed at high strain rates, a behaviour closely associated with cell wall collapse and the compression of internal gases.

### 4.3. Applicability of the Weibull Weakest-Link Theory

To assess the fitting performance of different size effect models for predicting the longitudinal compressive strength of bamboo, both the Weibull weakest-link theory Type I and Type II were employed in this study. Their predictive accuracy and parameter stability under various strain rates were compared using data presented in Table 3 and Table 5.

The fitting results demonstrate that both models accurately predicted the experimental outcomes across the full range of test conditions, with relative errors consistently maintained within 5%. Among them, the Type II model exhibited the best performance for medium-sized specimens (18 × 18 × 6 mm), achieving a minimum relative error of 0.10% and displaying no significant deviations across the majority of data points, thereby producing stable and reliable fitting curves. In comparison, while the Type I model also yielded acceptable error levels, its size effect parameters varied considerably with strain rate, indicating lower parameter stability.

From a practical modelling standpoint, the Type I model features a relatively simple formulation, making it suitable for preliminary analyses. However, its limited parameter stability and narrower range of applicability may constrain its use in complex scenarios. The Type II model, by contrast, offers higher fitting accuracy and effectively captures strength fluctuations induced by size variations, making it more appropriate for integration with strain rate models in comprehensive performance assessments.

In summary, the Type II model demonstrates slightly superior accuracy and parameter robustness compared to the Type I model and is therefore recommended for analysing size effects in the longitudinal compressive strength of *Chimonobambusa utilis*.

### 4.4. Applicability of the Strain Rate Model

According to the results, specimens with dimensions of 15 × 15 × 5 mm and 18 × 18 × 6 mm exhibited an n-value of 0.02, while the 21 × 21 × 7 mm specimen showed an n-value of 0.01. These findings indicate that, within the strain rate range of 10^−4^ s^−1^ to 10^−2^ s^−1^, *Chimonobambusa utilis* demonstrates low sensitivity of longitudinal compressive strength to strain rate variations, classifying it as a low-strain-rate-sensitive material.

Despite the relatively small strain rate sensitivity index, longitudinal compressive strength increased with higher loading rates. This trend suggests that dynamic loading suppresses microcrack initiation and propagation, thereby enhancing the overall load-bearing capacity. Further analysis revealed that strain rate responses varied among specimens of different sizes: smaller specimens (15 mm) exhibited a more pronounced strength increase at higher strain rates compared to larger specimens (21 mm). This indicates that specimen size plays a moderating role in the strain rate effect, with strain rate sensitivity being influenced by the specimen size effect and a trend of coupling between the two. This observation is consistent with the earlier two-way ANOVA results, which showed that the specimen size effect was significant, while the strain rate effect was not statistically significant but exhibited a tendency toward enhancement.

### 4.5. Validation and Improvement of the Coupled Model

Experimental results indicate that the longitudinal compressive strength of *Chimonobambusa utilis* is only weakly influenced by changes in strain rate, reinforcing its classification as a low-strain-rate-sensitive material. In contrast, specimen size exerts a more significant effect: as specimen size increases, longitudinal compressive strength decreases markedly. This trend underscores the dominant role of the specimen size effect in governing the longitudinal compressive performance of bamboo.

To better capture the coupling effect between specimen size and strain rate, an interaction term ${\left(D\cdot \dot{\varepsilon }\right)}^{\gamma }$ was introduced, leading to an improved coupled relationship, as expressed in Equation (16).

(16) $$\sigma =\sigma_{0}{\left(\frac{D}{D_{0}}\right)}^{-m}{\left(\frac{\dot{\varepsilon }}{\dot{\varepsilon_{0}}}\right)}^{n}{\left(\frac{D\cdot \dot{\varepsilon }}{D_{0}\dot{{\cdot \varepsilon }_{0}}}\right)}^{\gamma }$$

where *σ*_0_ is the reference strength; *D* is the specimen height; *D*_0_ is the reference size; $\dot{\varepsilon }$ is the applied strain rate; $\dot{\varepsilon_{0}}$ is the reference strain rate; *m* is the specimen size effect exponent; *n* is the strain rate sensitivity exponent; and *γ* is the interaction exponent.

Considering both model applicability and parameter stability, the specimen group with dimensions of 18 × 18 × 6 mm—demonstrating optimal size-effect fitting accuracy—was selected as the reference. The longitudinal compressive strength of this group at a strain rate of 10^−3^ s^−1^ (measured in MPa) was used as the baseline strength in the coupled model. Accordingly, the reference size was expressed in millimetres (mm), and the reference strain rate was explicitly defined.

Through nonlinear regression fitting of the experimental data, the interaction exponent *γ* was determined. Combined with the specimen size effect exponent *m* and the strain rate sensitivity exponent *n*, an improved coupled model was formulated, as shown in Equation (17).

(17) $$\sigma =70.17{\left(\frac{D}{18}\right)}^{-0.48}{\left(\frac{\dot{\varepsilon }}{10^{-3}}\right)}^{0.02}{\left(\frac{D\cdot \dot{\varepsilon }}{18\times 10^{-3}}\right)}^{-0.0032}$$

**Figure 14:**
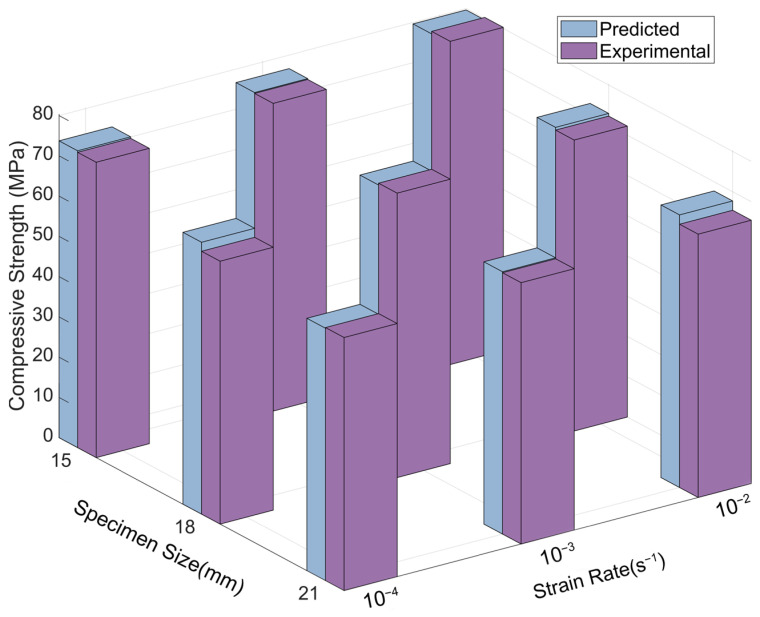
Comparison of predicted longitudinal compressive strength from the improved coupling model and experimental results.

Figure 14 presents the comparison between the predicted longitudinal compressive strength values from the improved coupled model and the experimentally measured values.

To assess the predictive accuracy of the improved model, a paired-sample *t*-test was conducted to compare the measured and predicted longitudinal compressive strength values. The analysis yielded a mean difference of −0.42 MPa, with no statistically significant difference between the two datasets (t(8) = −1.09, *p* = 0.31, 95% CI [−1.30, 0.46] MPa). The absence of significant deviation confirms that the model demonstrates strong predictive capability under the current experimental conditions, with predicted values closely aligning with the measured data.

To further verify the accuracy of the modified coupling model, longitudinal compression tests were conducted on 12 × 12 × 4 mm specimens under different strain rates. Table 8 compares the experimentally obtained longitudinal compressive strengths and the values predicted by the model.

The experimental results in Table 8 are in close agreement with the predicted values, with only minor deviations, thereby validating the effectiveness and reliability of the improved model in predicting the mechanical performance of small-diameter bamboo. These new experimental data further support the rationality of the size–strain rate coupling effect model proposed in this study and provide a theoretical basis for its broader application in practical engineering.

### 4.6. Limitations

(1) Limitations of Experimental Conditions and Applicability Scope

*Chimonobambusa utilis* is a small-diameter bamboo species, resulting in correspondingly small specimen dimensions (see Figure 1). The investigated strain rate range primarily falls within the low to intermediate region and does not include extreme dynamic conditions such as high-velocity impact. As a result, the applicability of both the experimental findings and the derived model parameters is restricted to the present test conditions. Additional validation using larger specimen sizes and broader strain rate ranges is necessary to extend model generalizability.

(2) Limitations in Model Construction

The improved coupling model, based on the Weibull weakest-link theory and strain rate sensitivity formulation, relies on regression analysis of experimental data for parameter identification, classifying it as an empirical model. Although the model effectively captures the influence of specimen size and strain rate on mechanical performance, it does not explicitly account for the anisotropic characteristics of bamboo’s microstructure—such as vascular bundle distribution and fibre orientation—which may influence predictive accuracy and broader applicability.

## 5. Conclusions

The longitudinal compressive strength of small-diameter *Chimonobambusa utilis* was systematically analysed under varying specimen sizes and strain rates, and the results indicated that the specimen size effect was the dominant factor, while the strain rate played a moderating and enhancing role, with a trend-level interaction observed between them. For the first time, a size–strain rate coupling prediction model applicable to natural small-diameter bamboo was developed, as expressed below:

(18) $$\sigma =70.17{\left(\frac{D}{18}\right)}^{-0.48}{\left(\frac{\dot{\varepsilon }}{10^{-3}}\right)}^{0.02}{\left(\frac{D\cdot \dot{\varepsilon }}{18\times 10^{-3}}\right)}^{-0.0032}$$

In the Equation, *σ* denotes the strength, *D* represents the height, and $\dot{\varepsilon }$ refers to the strain rate controlled during the test.

From a modelling perspective, the Weibull weakest-link theory Type II was innovatively employed to characterise the specimen size effect and was nonlinearly coupled with a power-law strain rate formulation. An interaction exponent was further introduced to construct an enhanced size–strain rate coupling model. For the first time, relevant parameters were quantitatively defined for small-diameter bamboo. This model offers a theoretical foundation for structural design and strength assessment in green building applications and provides a methodological basis for future modelling of engineered, large-diameter bamboo materials.

Due to limitations in specimen dimensions and strain rate scope, the current model relies heavily on fitted experimental data, which restricts its broader applicability. Future research will focus on fabricating engineered bamboo materials from *Chimonobambusa utilis* with larger dimensions and incorporating microstructural factors such as anisotropy to improve the generality and predictive capability of the model.

## Figures and Tables

**Table 1:** Loading speeds corresponding to target strain rates.

Strain Rate (s)	15 mm (mm/min)	18 mm (mm/min)	21 mm (mm/min)
10^−4^	0.09	0.108	0.126
10^−3^	0.9	1.08	1.26
10^−2^	9	10.8	12.6

**Table 2:** Basic mechanical properties.

Strain Rate (s)	Specimen Size (mm)	Longitudinal Compressive Strength Parallel to Grain (MPa)	Poisson’s Ratio	Elastic Modulus (N/mm^2^)	Peak Strain (%)	Standard Deviation (MPa)	Coefficient of Variation
10^−4^	15 × 15 × 5	73.35	0.15	3148.33	2.34%	6.50	0.09
	18 × 18 × 6	65.28	0.17	2646.44	2.46%	9.73	0.15
	21 × 21 × 7	62.84	0.15	2944.60	2.21%	3.56	0.06
10^−3^	15 × 15 × 5	76.42	0.13	3847.17	2.08%	2.94	0.66
	18 × 18 × 6	70.63	0.15	3016.81	2.35%	2.91	0.04
	21 × 21 × 7	64.90	0.16	2303.23	2.87%	2.41	0.04
10^−2^	15 × 15 × 5	80.27	0.12	2389.59	3.18%	1.84	0.02
	18 × 18 × 6	72.26	0.15	3026.95	2.47%	3.20	0.04
	21 × 21 × 7	65.39	0.17	3281.20	1.99%	1.15	0.02

The standard deviation and coefficient of variation are calculated based on the longitudinal compressive strength.

**Table 3:** Fitting results based on the Weibull weakest-link theory Type I.

Strain Rate (s)	Specimen Size (mm)	Measured Value (MPa)	Predicted Value (MPa)	Error (MPa)	Relative Error (%)	$\mathrm{Size}\;\mathrm{Effect}\;\mathrm{Parameter}\;S_{\tau }$	R^2^
10^−4^	15 × 15 × 5	73.35	72.62	0.73	−0.99	0.23	0.945
	18 × 18 × 6	65.28	66.72	−1.44	2.20		
	21 × 21 × 7	62.84	62.10	0.74	−1.17		
10^−3^	15 × 15 × 5	76.42	76.65	−0.23	0.30	0.24	0.995
	18 × 18 × 6	70.63	70.17	0.46	−0.65		
	21 × 21 × 7	64.90	65.13	−0.23	0.35		
10^−2^	15 × 15 × 5	80.27	80.42	−0.15	0.18	0.30	0.999
	18 × 18 × 6	72.26	71.97	0.29	−0.40		
	21 × 21 × 7	65.39	65.53	−0.14	0.22		

**Table 4:** Fitted parameters derived from linear regression based on the Weibull weakest-link theory Type II.

Strain Rate (s)	$\sigma_{0}$ (MPa)	*m*	R^2^	Error (MPa)	Relative Error (%)
10^−4^	66.72	0.47	0.945	0.97	1.46
10^−3^	70.17	0.48	0.995	0.43	0.61
10^−2^	71.97	0.61	0.999	3.03	4.18

**Table 5:** Fitting results based on the Weibull weakest-link theory Type II.

Strain Rate (s)	Specimen Size (mm)	Measured Value (MPa)	Predicted Value (MPa)	Error (MPa)	Relative Error (%)
10^−4^	15 × 15 × 5	73.35	72.62	−0.73	1.00
	18 × 18 × 6	65.28	66.72	1.44	2.21
	21 × 21 × 7	62.84	62.10	−0.74	1.18
10^−3^	15 × 15 × 5	76.42	77.07	0.65	0.85
	18 × 18 × 6	70.63	70.56	−0.07	0.10
	21 × 21 × 7	64.90	65.48	0.58	0.89
10^−2^	15 × 15 × 5	80.27	77.06	−3.21	4.00
	18 × 18 × 6	72.26	68.97	−3.29	4.55
	21 × 21 × 7	65.39	62.79	−2.60	3.98

**Table 6:** Fitted strain rate sensitivity parameters for specimens of different sizes.

Specimen Size (mm)	$\sigma_{0}$ (MPa)	*n*
15 × 15 × 5	73.24	0.021
18 × 18 × 6	65.96	0.023
21 × 21 × 7	63.11	0.009

**Table 7:** Comparison between predicted and measured values based on the strain rate sensitivity model.

Specimen Size (mm)	Strain Rate (s)	Measured Value (MPa)	Predicted Value (MPa)	Error (MPa)	Relative Error (%)
15 × 15 × 5	10^−4^	73.35	73.24	−0.11	0.15
	10^−3^	76.42	76.63	0.21	0.27
	10^−2^	80.27	80.17	−0.10	0.12
18 × 18 × 6	10^−4^	65.28	65.96	0.68	1.04
	10^−3^	70.63	69.33	−1.30	1.84
	10^−2^	72.26	72.88	0.62	0.85
21 × 21 × 7	10^−4^	62.84	63.11	0.27	0.43
	10^−3^	64.90	64.37	−0.53	0.82
	10^−2^	65.39	65.65	0.26	0.40

**Table 8:** Comparison between Experimental Values and Model Predictions.

Specimen Size (mm)	Strain Rate (s)	Measured Value (MPa)	Predicted Value (MPa)	Error (MPa)	Relative Error (%)
12 × 12 × 4	10^−4^	80.56	82.12	1.56	1.93
	10^−3^	83.92	85.36	1.44	1.71
	10^−2^	89.74	88.72	−1.02	1.13

## Data Availability

The raw data supporting the conclusions of this article will be made available by the authors on request.
